# Editorial: Tissue crosstalk in obesity and diabetes: a focus on skeletal muscle

**DOI:** 10.3389/fendo.2025.1724016

**Published:** 2025-11-10

**Authors:** Ana Luísa De Sousa-Coelho, M. Dulce Estêvão, Mary Elizabeth Patti, Carles Lerin

**Affiliations:** 1 Escola Superior de Saúde, Universidade do Algarve, Faro, Portugal; 2 Algarve Biomedical Center Research Institute (ABC-Ri), Faro, Portugal; 3 Joslin Diabetes Center, Boston, MA, United States; 4 Harvard Medical School, Boston, MA, United States; 5 Institut de Recerca Sant Joan de Déu, Barcelona, Spain; 6 School of Medicine, University of Vic - Central University of Catalonia, Vic, Spain; 7 Centro de Investigación Biomédica en Red de Diabetes y Enfermedades Metabólicas Asociadas (CIBERDEM), Instituto de Salud Carlos III, Madrid, Spain

**Keywords:** metabolism, crosstalk, skeletal muscle, obesity, diabetes, extracellular vesicles, myokines

In the complex biological system of higher organisms, the maintenance of metabolic homeostasis requires intricate crosstalk among different tissues and organs. Such inter-organ communication, including classical hormones, other peptides, and extracellular vesicles (EVs), allows one tissue to affect metabolic pathways in a distant tissue. Dysregulation of this communication contributes to human pathologies, including obesity, diabetes, liver diseases, and certain cancers.

This Research Topic aimed to shed light onto the complex tissue crosstalk underlying metabolic diseases, specifically obesity and type 2 diabetes (T2D). It highlights the latest scientific evidence exploring such interactions among different tissues, with a specific focus on the role of skeletal muscle and its secretion of myokines and EVs that contribute to the regulation of metabolism in liver, adipose tissue, and other organs.

The language of muscle-based communication is largely spoken through myokines. Kurose et al. provide a nuanced view of the Myostatin (MSTN) and Follistatin (FST) response to weight loss in obesity, demonstrating that their dynamics are not merely a function of weight change but are also influenced by physical activity and the degree of metabolic improvement. Additionally, Bunn et al. present the therapeutic promise of targeting this system, revealing that MSTN inhibition, when combined with insulin, confers superior benefits for both muscle mass and trabecular bone in a female mouse model of type 1 diabetes, underscoring the muscle-bone crosstalk. The study by Ji et al. highlights Prosaposin (PSAP) and Ependymin-related protein 1 (EPDR1), recently identified as circulating factors secreted by muscle and/or adipose tissue (1, 2). The authors found that the levels of these factors are elevated in T2D and strongly correlate with insulin resistance, proposing them as potential new biomarkers and therapeutic targets.

MG53/TRIM72 has emerged as a promising target in regenerative medicine due to its significant ability to repair cell membranes and exert anti-inflammatory effects in a variety of tissues, including skeletal muscle. However, several studies have raised concerns that MG53 may function as a diabetogenic myokine by disrupting insulin signaling (3). In this study, Lee et al. demonstrate that MG53 does not bind to the insulin receptor at physiologic concentrations and does not impair insulin-stimulated Akt phosphorylation in cultured myotubes and muscle tissues. This discovery may facilitate the progression of MG53-based therapies for acute or chronic tissue injuries without the concern for induction of insulin resistance.

Adipose infiltration in skeletal muscle impairs muscle health and function. Adipocytes can infiltrate within muscle fibers and secrete both lipotoxic molecules and pro-inflammatory cytokines. This typically results in mitochondrial dysfunction, insulin resistance, and impaired repair mechanisms, potentially leading to accelerated sarcopenia (4). Zhu et al.**’**s review summarizes the available information on the abnormal accumulation of adipocytes in non-adipose tissues and on the cellular mechanisms by which intramuscular adipose can contribute to muscle degeneration, including lipotoxicity, inflammatory signaling, impaired stem-cell fate, and extracellular matrix remodeling. Furthermore, the authors provide some therapeutic targets to prevent or reverse fatty infiltration and alleviate age-related musculoskeletal disorders.

Bariatric and metabolic surgery (BMS) is an effective treatment for obesity and T2D, yielding sustained improvement or even remission of T2D and reduced risk for cardiovascular disease and other complications. However, weight loss induced by either BMS or highly effective obesity medications can also promote loss of fat-free mass (5, 6), which may compromise glucose metabolism and muscle strength. Orioli et al. provide a comprehensive review exploring how changes in certain myokines after BMS may be related to both loss of muscle mass and changes in glucose metabolism. The authors report that some circulating myokines increase after BMS, such as Fractalkine (CX3CL1), Fibroblast Growth Factor 21 (FGF21) and Myonectin (CTRP15), while others decrease, such as brain-derived neurotrophic factor (BDNF), MSTN, Apelin (APLN) and Secreted protein acidic and rich in cystein (SPARC). It is important to acknowledge that it is challenging to establish skeletal muscle as the main source of these myokines. Orioli et al. also pointed conflicting results in the literature, which may be due to the different time points after BMS analyzed, and/or differences in the specific characteristics of the populations in each study.

Considering how distant tissues may communicate, produced and released by cells into the extracellular environment, EVs promote interactions within distal organs. Although to be fully characterized, their cargo may include different kinds of biomolecules (i.e., cytokines, microRNAs, lipids) (7). Two independent reviews deepen the knowledge on specific aspects of EVs research, either focusing in the role of EVs in the regulation of glucose and lipid metabolism, or exploring the relationship between EVs and T2D complications. Zhang et al. discuss how adipose tissue macrophages (ATMs)-derived EVs may mediate the communication between macrophages and adipocytes in individuals with metabolic diseases, highlighting that the extended knowledge on such crosstalk may be explored for future clinical applications. Importantly, ATMs-EVs may be coordinating the interactions between adipose tissue and other key metabolic tissues, such as skeletal muscle. Liu et al. reinforce that EVs should be considered as potential therapies. In this detailed review, the authors describe the known mechanisms linking EVs and T2D, namely their main cargo and tissue sources. For many diabetic complications, such as nephropathy, retinopathy, cardiomyopathy, there is evidence for the involvement of EVs in their pathogenesis.

Considering the brain-muscle-adipose tissue crosstalk, an original article investigated the relevance of low skeletal muscle mass alongside central obesity on cognitive impairment (CI) (Zhai et al.). Using data from a cross-sectional study, the authors estimated basal metabolic rate (BMR) and employed a mediation analysis model. Lower BMR-related parameters were identified in T2D patients with CI, suggesting that BMR may serve as a biomarker or screening parameter for cognitive risk in T2D. Given the recent definition of clinical obesity focused on complications of obesity (8), it highlights that energy expenditure, rather than body composition alone, may be an important factor to improve our understanding of cognitive impairment in T2D.

Finally, a conceptual breakthrough is provided by Song et al., who offer compelling evidence for a sex-specific gut-muscle axis. The authors demonstrate that metformin’s effects on skeletal muscle are sexually dimorphic and are associated with distinct, sex-dependent changes in gut microbiota, plasma short-chain fatty acids (SCFAs), and muscle SCFA receptor expression. This work highlights the gut microbiome as a significant regulator of muscle physiology and underscores an important recurring theme in research: sex differences. The deliberate focus on female models and the sex-differentiated outcomes, is a crucial reminder that the rules of tissue crosstalk may not be universal.

Collectively, these articles present a cohesive picture of a sophisticated, multi-organ dialogue with skeletal muscle at its core. From the complex regulation of myokines and EVs to the emerging influence of the gut microbiome, these findings move us beyond simplistic models and pave the way for more precise and personalized strategies to treat metabolic diseases.
